# Evaluation of a new emergency department avoidance model of care, the Cancer Urgent Assessment Clinic, in response to the COVID-19 pandemic

**DOI:** 10.1186/s44201-022-00011-8

**Published:** 2022-10-03

**Authors:** Corrine Haugstetter, Robert Mason, Jasotha Sanmugarajah, H. Laetitia Hattingh

**Affiliations:** 1 Cancer, Blood and Respiratory Medicine, Gold Coast Health, Southport, QLD 4215 Australia; 2 Medical Services, Clinical Governance and Research, Gold Coast Health, Southport, QLD 4215 Australia; 3 School of Pharmacy and Medical Sciences, Griffith University, Southport, QLD 4222 Australia

**Keywords:** Cancer toxicities, Emergency oncology, Emergency department avoidance, Telephone triage

## Abstract

**Introduction:**

The Cancer Urgent Assessment Clinic (CUAC) was an emergency department (ED) avoidance/unscheduled model of care implemented in response to the COVID-19 pandemic. The aim was to reduce the risk of COVID-19 exposure and infection by providing an alternative to ED for cancer patients while undergoing anticancer treatments.

**Methods:**

The clinic incorporated a telephone triage process and face-to-face appointments 8am to 8pm, 7 days per week. CUAC operated between 23 March '20 and 31 July '20, led by a nurse practitioner candidate, oncology registrars, cancer nurse specialists, and overseen by oncology consultants. Evaluation followed a mixed-methods approach through (1) analysis of CUAC patient data, (2) comparison of ED cancer patient presentation data from a previous period (23 March 2019–31 July 2019), and (3) a patient survey.

**Results:**

In total, 400 patients were telephone triaged via CUAC, with 166 recorded as having avoided ED. There was a reduction in the number of cancer patient admissions to the ED short stay unit during the clinic period compared with the same time-period in 2019: 130 vs. 234, associated with 615 fewer hours. Patient satisfaction was positive particularly regarding ease of access, time to treatment, confidence in assessment and treatment of cancer-related concerns, and likelihood of presenting to hospital when unwell during the pandemic.

**Discussion:**

While initially being implemented to reduce the risk of COVID-19 exposure, this evaluation demonstrated the CUAC model was an efficient and potentially cost-saving model of care for the management of cancer patients with mild to moderate severity of disease and treatment-related concerns.

## Background

There is a global increase in demand for cancer services: the World Health Organization in 2018 reported 18.1 million people around the world had cancer and estimated the figure to double by 2040 [1]. In the Australian Institute of Health and Welfare estimated, there were 143,205 new cancer diagnoses in 2019, expected to rise to approximately 145,483 in 2020 [2]. Approximately 90% of all new cancer diagnoses occur in those aged 50 years and above, which is associated with a higher rate of comorbidity and subsequent disease and treatment-related complications [2–4]. Concurrently, there have been significant improvements in cancer screening, diagnosis, treatment, and subsequently survival following initial diagnosis over the past 20 years: from a 50% 5-year relative survival in 1986–1990 to 69% in 2011–2015 [2, 5]. It is also due to the development of new targeted treatments for some cancers resulting in more people living with cancer and undergoing systemic anti-cancer treatments (SACT) for longer [2].

Patients with cancer commonly experience distressing symptoms or side effects from their disease and/or SACT that prompt them to attend emergency departments (EDs) for assessment and treatment. Several studies demonstrate that ED presentation rates were higher for cancer patients compared with the general population, although it has been recognized that further research is required to define the ED utilization and patterns of presentation in this population [6–10]. A retrospective analysis of 2009–2012 data from four hospitals in Victoria, Australia, showed that cancer patients who presented to EDs were of a higher acuity, remained in EDs for longer, required admission more frequently, and the resulting inpatient admissions recorded a longer length of stay (LoS) as well as patients having higher rate of representation when compared with non-cancer patients [8].

Patients with cancer, particularly those undergoing immunosuppressive SACT, are susceptible to infections due to the immunocompromised state that arises from the malignancy itself and/or treatment [11]. There is evidence supporting improved outcomes when there is access to health professionals who have specialist experience with cancer and treatment-related toxicity management, such as an acute oncology service, as this provides more timely and effective care and results in reduced ED presentation and avoidable inpatient admissions [12–14]. Alternative models of care that facilitate treatment while avoiding ED presentation and inpatient admission, such as ambulatory care models and home-based services, are showing to be cost-effective and safe adjuncts [15, 16]. There is also growing evidence for specialist oncology nurse-led triage services [17, 18]. A United Kingdom (UK) study that involved a newly introduced nurse-led telephone triage system at an oncology service showed appropriate triaging and high patient satisfaction [19]. An Australian study of a nurse practitioner-led model of care consisting of a telephone helpline, urgent assessment clinic, and rapid day treatment consultation service resulted in reduced ED presentations and better symptom management [20].

There were concerns at the start of the COVID-19 pandemic that cancer patients who contracted COVID-19 would have a higher risk of rapid clinical deterioration and severe adverse events including intensive care admission and subsequent invasive ventilation [11]. It was recognized that patients undergoing immunosuppressive SACT were at higher risk for COVID-19 infection [21–23]. Hence, a need was identified for an alternative model of care during the pandemic to facilitate the management of cancer patients. The Gold Coast Hospital and Health Service (GCHHS) located in Queensland, Australia, developed an alternative pathway for medical oncology patients to access unscheduled care during the pandemic to avoid ED presentations and reduce unnecessary inpatient admissions. Considering the literature and an existing model of care at an Australian Capital Territory Health Service [24, 25], the Cancer Urgent Assessment Clinic (CUAC) was implemented on 23 March 2020.

The CUAC service provided timely assessment and treatment for patients with cancer experiencing acute disease or treatment-related complications. The overall aim of the service was to reduce cancer patient exposure to COVID-19 infection by offering an alternative to ED presentation and therefore indirectly reducing contact with COVID-19-positive patients, expedite sub-specialty assessment and admission when required, reduce length of hospital stay, and provide real-time support and advice to primary health professionals (e.g., general practitioners (GPs)). An evaluation of the CUAC service was conducted to inform future service planning and potential integration into standard practice.

## Methods

The evaluation of the CUAC followed a mixed methods approach through an analysis of CUAC patient data, a comparison of cancer patient ED presentations pre- and post-implementation, and a patient survey. Ethics approval was granted by the GCHHS Human Research Ethics Committee (LNR/2020/QGC/68515). The TIDieR checklist was followed to evaluate the intervention [26].

The objectives of the evaluation were as follows:

1. Evaluate the presentation patterns of cancer patients who experienced treatment or disease-related complications while undergoing outpatient SACT who were referred to the CUAC
2. Compare the ED utilization of cancer patients undergoing SACT pre- and post- implementation of the CUAC
3. Explore cancer patients’ experience and feedback on the CUAC model of care

### Setting

The CUAC was implemented at the Gold Coast University Hospital (GCUH), a tertiary hospital that is part of GCHHS. GCUH has a designated Cancer and Blood Disorders Outpatient Department with specialist medical oncology and hematology teams. Multidisciplinary teams consist of medical specialists, specialist nurses, and allied health professionals. The department provides on average 124 medical reviews/day during weekdays and administration of SACT to approximately 70 patients/day on Mondays to Saturdays. Access to advice has traditionally occurred solely via telephone contact with the chemotherapy day unit or the cancer nurse specialist team during business hours, or the inpatient hematology/oncology unit after hours. It was standard practice for patients who required urgent assessment to be directed to present via the ED due to clinic capacity. Clinic appointments are booked 2–3 weeks in advance, limiting availability for emergency appointments.

### Intervention

The CUAC was a new nurse-led model of care that was trialed 23 March 2020 to 31 July 2020. Prior to this model, there was no centralized or standardized method for assessing patient-initiated contact regarding treatment or disease-related concerns. Patients directed their concerns to their allocated cancer nurse specialist which led to inconsistency in access and management. The CUAC incorporated a telephone triage line with access to a face-to-face clinic for assessment and treatment and was operated from the Cancer and Blood Disorders Day Unit during business hours (8am to 4pm). It was managed afterhours from the Haematology Oncology Inpatient Unit. There was access to either treatment chairs or hospital beds as required and clinicians could order required investigations (pathology via the hospital’s core laboratory, radiology including X-ray, ultrasound, computed tomography, and magnetic resonance imaging) to assess patients. Treatments included intravenous infusions, medication administration, blood product administration, and paracentesis (following ultrasound marking). The maximum capacity of the service was two physical spaces at any given time. The service was provided on an 8am to 8pm basis, 7 days per week. The telephone triage line was manned by clinical nurse consultants (CNCs) from the cancer nurse specialist team on a rotational basis during business hours, and the inpatient ward team leader (clinical nurse (CN) or registered nurse (RN)) after hours. The CUAC was staffed primarily by a nurse practitioner candidate during business hours with support from an RN from the chemotherapy day unit and by a medical officer with nursing support from an inpatient unit RN after hours. The medical consultants provided oversight and clinical support on a rotational basis. The CUAC triage guidelines followed the United Kingdom (UK) Oncology Nursing Society (UKONS) Oncology Hematology 24-H Helpline, Rapid Assessment and Access Toolkit—Australia [27], that was based on the UK guidelines [18], to assess symptom/toxicity severity and utilized the validated escalation guidelines within the toolkit. The UKONS telephone triage guidelines utilize a “traffic light” system of toxicity grading, where urgent face-to-face assessment is required when one toxicity is graded red, or two or more toxicities are graded as yellow; and non-urgent assessment or self-care advice with planned follow-up within 24 h is required if one toxicity is graded as yellow [27].

#### CUAC patient eligibility

Inclusion criteria were adult cancer patients with a solid tumor diagnosis that contacted or were referred to the CUAC service who were under the care of a treating medical oncologist at GCHHS at the time, undergoing SACT at GCHHS or had completed SACT within 6 weeks of presentation, experiencing disease or treatment related complications. Patients who were *not eligible* to receive advice or attend the CUAC were patients with a hematological malignancy as these patients already had an existing model for urgent assessment and management; OR patients who presented with complaints not related to their cancer diagnosis or treatment.

### Data sources

#### CUAC patient data

CUAC patient data collected included the date of CUAC service contact, patient identifiers, cancer diagnosis and treatment details; reason for contact/presenting complaint(s); severity of presenting complaint(s) based on UKONS Oncology Haematology Rapid Assessment and Access Toolkit [27] severity grading scale; method of referral (patient-initiated contact, GP, ED, clinician); action taken following triaging assessment; whether face-to-face assessment occurred and date of assessment; outcome of assessment (admission or discharge); and whether representation(s) occurred.

#### Cancer patient ED presentation and admission data

Data was extracted from the hospital electronic medical record systems for the duration of the CUAC period of operation (23 March 2020 to 31 July 2020) and the corresponding time-period in the previous year (23 March 2019 to 31 July 2019). Data extracted were any ED presentations by cancer patients that followed the same inclusion/exclusion criteria as those applied for CUAC patient eligibility.

#### Patient survey

Patients who contacted the CUAC were invited to participate in a survey to explore their experiences and satisfaction with the CUAC model of care. A patient information sheet was attached to the survey to explain the purpose of the survey and that participation was voluntary. Completion of the survey was regarded as consent.

### Participant recruitment

Patients who physically attended the CUAC service for assessment and management were approached at their following medical oncology outpatient review to inform them about the study and provided the information sheet and survey. Those who had already been discharged from medical oncology (due to completion of treatment, relocation or transfer of care to another specialty) or did not have a review booked within 4 weeks following their CUAC visit received the survey with a reply-paid envelope via post, excluding patients who were deceased or those who were unable to complete an English language survey. A research assistant made follow-up phone calls a few weeks after patients received the survey.

### Development of survey

The patient survey was developed considering the literature and study objectives. Questions focused on participants’ perceived safety and impact of the COVID-19 pandemic on their confidence in accessing emergent care via the CUAC with a small number of questions from the European Organization for Research and Treatment of Cancer satisfaction with cancer care core questionnaire (EORTC PATSAT-C33) [28]. The survey incorporated a question on whether patients had previously attended the ED for treatment related toxicity, prior to the implementation of the CUAC, to allow for comparison of experiences of the different models of care. The survey consisted of a combination of question formats, including 5-point Likert-type questions. Feedback on potential improvements was also requested through open-ended questions.

The survey was reviewed and validated by the cancer nurse specialist and medical teams and consumer engagement committee representative. Recommended changes to wording were incorporated.

### Data analysis

Data was entered into a Microsoft Excel© spreadsheet and survey data into SPSS® for analysis. Likert scales were coded as 1 = poor, 2 = fair, 3 = good, 4 = very good, and 5 = excellent. Standard descriptive statistics were used to summarize numbers and percentages.

## Results

### CUAC referrals

In total, 400 patient presentations were triaged by the CUAC service between 23 March 2020 and 31 July 2020 (some patients presented more than once). The primary method of referral to the CUAC was patient/family member-initiated contact via the telephone triage line (220/400; 55.0%), followed by referral by the CNCs/CNs of the cancer nurse specialist team (97/400; 24.3%), 13% (52/400) referred by the treating team following outpatient clinical review (e.g., unwell presentations, incidental findings on routine scans, or pathology results), 6% (24/400) after presenting to ED, and 1.8% (7/400) referred by GPs.

As a result of the telephone triage process, 218/400 patients (54.5%) were evaluated as requiring urgent face-to-face assessment, 49/400 patients (12.3%) were evaluated as requiring a non-urgent assessment, and two additional patients that did not meet triage severity criteria but presented in person to the clinic (2/400; 0.5%) were managed by the CUAC, resulting in a total 246 patients attending the CUAC for in-person assessment and management. Of those 246 patients that were managed in the CUAC clinic, 129 (52.4%) were directly admitted to the inpatient unit for ongoing management, whilst 117 (47.6%) were able to be discharged home. There was one medical emergency team activation for a patient that became unstable (de-saturated) who was transferred to the intensive care unit for ongoing management following assessment by the team.

Of the 218 patients evaluated as requiring urgent assessment, 76.1% (166/218) were assessed and managed by the CUAC and therefore potentially avoided ED presentation. Overall, 23.9% (52/218) triaged patients were either directed to ED due to meeting criteria for COVID-19 testing and isolation or assessed as clinically unstable (23/218; 10.6%), directed to ED due to the CUAC reaching treatment space capacity (17/218; 7.8%), declined to attend for assessment despite recommendation to do so (10/218; 4.6%), or not eligible to attend CUAC (2/218; 0.9%). Those that declined review were followed-up with a telephone call to reassess symptoms within 24 h. An additional 31 patients were referred to the CUAC following direct ED presentation with a limited evaluation or triage.

### CUAC patient data

The 246 patients who were managed by the CUAC ranged from 22 years to 87 years (median 62 years), with females accounting for 146/246 presentations (59.3%) and males accounting for 100/246 presentations (40.7%). The patients’ cancer diagnoses were representative of the medical oncology patient population managed at the GCUH: lung cancer (20.7%), colorectal cancer (18.7%), breast cancer (17.9%), upper gastrointestinal/hepatobiliary cancer (15.4%), gynecological cancer (9.8%), urogenital cancer (6.1%), melanoma (5.3%), head and neck cancer (4.5%), cancer of unknown primary (1.2%), and brain cancer (0.4%); with 152/246 having metastatic disease (61.8%).

The 246 patients who were managed by the CUAC mostly presented with general and systemic complaints of fever and pain. Table 1 provides a summary of the primary or most severe presenting complaints for all presentations, based on the UKONS toxicity grading, with Table 2 a summary of the primary or most severe presenting complaints for the 129 patients who were admitted the inpatient unit for ongoing management.

**Table 1:** Primary or most severe presenting complaint for all Cancer Urgent Assessment Clinic presentations (based on UKONS toxicity grading [27])

Primary presenting complaint	Total; ***n***=246, ***n*** (%)	UKONS red grading, ***n*** (%)
**General/systemic**		
Pain	51 (20.7%)	27 (25.7%)
Fever—on systemic treatment	28 (11.4%)	28 (26.7%)
Infection	12 (4.9%)	1 (0.9%)
Performance status	10 (4.1%)	4 (3.8%)
Fatigue	4 (1.6%)	1 (0.9%)
	**105 (42.7%)**	**61/105 (58.1%)**
**Gastrointestinal/urinary**		
Diarrhea	15 (6.1%)	5 (2.0%)
Urinary disorder	11 (4.5%)	5 (2.0%)
Nausea	9 (3.7%)	2 (0.8%)
Vomiting	8 (3.3%)	2 (0.8%)
Mucositis	6 (2.4%)	3 (1.2%)
Constipation	5 (2.0%)	3 (1.2%)
Anorexia	1 (0.4%)	0 (0.0%)
	**55 (22.4%)**	**20/55 (36.3%)**
**Cardiovascular**		
Dyspnea	25 (9.8%)	7 (24.1%)
Chest pain	2 (0.8%)	2 (6.9%)
Bleeding	2 (0.8%)	2 (6.9%)
	**29 (22.4%)**	**11/29 (37.9%)**
**Central nervous system**		
Neurosensory/motor disturbance	10 (4.1%)	4 (30.8%)
Confusion/cognitive disturbance	3 (1.2%)	0 (0.0%)
	**13 (5.3%)**	**4/13 (30.8%)**
**Skin/eyes**		
Rash	4 (1.6%)	2 (25.0%)
Ocular/eye problems	2 (0.8%)	0 (0.0%)
Extravasation	1 (0.4%)	1 (12.5%)
Palmar plantar syndrome	1 (0.4%)	0 (0.0%)
	**38 (3.3%)**	**3/8 (3.7.5%)**
**Other**^**a**^		
	**36 (14.6%)**	**17/36 (47.2%)**

^a^ Other includes symptomatic abdominal ascites, concerns with central venous access devices or gastrostomy tubes, planned follow-up, symptomatic peripheral edema, urgent recall due to findings on routine labs/scans (e.g., incidental finding of pulmonary embolus, deranged liver function tests or electrolytes, positive blood cultures)

**Table 2:** Primary or most severe presenting complaint for patients admitted to the inpatient unit (based on UKONS toxicity grading) [27]

Primary presenting complaint	Total; ***n***=129 ***n*** (%)	UKONS red grading
**General/systemic**		
Pain	25 (19.4%)	15 (27.8%)
Fever—on systemic treatment	21 (16.3%)	21 (38.9%)
Nausea	5 (3.9%)	2 (3.7%)
Performance status	4 (3.1%)	2 (3.7%)
Infection	3 (2.3%)	1 (1.9%)
Fatigue	1 (0.8%)	0 (0.0%)
	**54 (41.9%)**	**39/54 (72.2%)**
**Gastrointestinal/urinary**		
Diarrhea	11 (8.5%)	5 (14.7%)
Vomiting	6 (4.7%)	2 (5.9%)
Urinary disorder	5 (3.9%)	1 (2.9%)
Constipation	5 (3.9%)	3 (8.8%)
Anorexia	1 (0.8%)	0 (0.0%)
Mucositis	1 (0.8%)	1 (2.9%)
	**34 (26.4%)**	**14/34 (41.1%)**
**Cardiovascular**		
Dyspnea	14 (10.9%)	4 (22.2%)
Chest pain	2 (1.6%)	2 (11.1%)
Bleeding	2 (1.6%)	2 (11.1%)
	**18 (13.9%)**	**8/18 (44.4%)**
**Central nervous system**		
Neurosensory/motor disturbance	7 (5.4%)	4 (40.0%)
Confusion/cognitive disturbance	3 (2.3%)	0 (0.0%)
	**10 (7.8%)**	**4/10 (40.0%)**
**Skin/eyes**		
Ocular/eye problems	1 (0.8%)	0 (0.0%)
	**1 (0.8%)**	**0/1 (0.0%)**
**Other**^**a**^		
	**12 (9.3%)**	**9/12 (75.0%)**

^a^ Other includes symptomatic abdominal ascites, concerns with central venous access devices or gastrostomy tubes, urgent recall due to findings on routine labs/scans (e.g., incidental finding of pulmonary embolus, deranged liver function tests or electrolytes, positive blood cultures)

### Patient with cancer ED presentation and admission data

There was only a small reduction in the number of CUAC eligible patients with cancer presenting to the ED during the period of CUAC operation compared with the same period in 2019: 315 presentations vs. 347 presentations. There was however a significant reduction in the number of CUAC eligible patients with cancer admissions to the ED short stay unit during the period of CUAC operation compared with the same period in 2019: 130 admissions vs. 234 admissions in the previous period, which is associated with a reduction in ED short stay hours of 614.57 h (710.3 vs. 1324.87). The ED short stay is an area that provides short-term admission between one and 24 h for a select group of patients, e.g., low risk for clinical deterioration, not expected to need care for > 24 h, aimed to provide cost-efficient short-stay admissions. The unit is designated and designed for the short-term treatment, observation, assessment, and re-assessment of patients initially triaged and assessed in the ED.

### Patient survey

A total of 115 patients were invited to participate in the survey with 60 surveys completed, a 52% response rate. Responses to rating of the CUAC service and the clinical team members were overall very positive (Table 3).

**Table 3:** Patient’s rating summaries of Cancer Urgent Assessment Clinic

Statement	^**a**^Mean score
***How would you rate the Cancer Urgent Assessment Clinic (CUAC) in terms of:***	
Ease of access to the service?	4.48
How quickly you were able to book an appointment to attend for an assessment?	4.37
On arrival at the hospital, how quickly you were assessed and treated?	4.48
Overall experience of the CUAC for assessment and management of cancer or treatment related concerns?	4.73
Satisfaction of the outcome from your presentation to the CUAC?	4.66
***How would you rate the CUAC clinical team in terms of:***	
Their knowledge of your cancer diagnosis and treatment history?	4.63
The attention they gave to your physical symptoms?	4.68
Their thoroughness in treating your physical symptoms?	4.70
The information they gave you about your illness?	4.62
The information they gave you about your medical tests and treatment?	4.62
Their taking your preference into account when discussing options for treatment (if different options were presented)?	4.58
The comfort and support they gave you?	4.73
The advice they gave you on managing your physical symptoms?	4.67
Your confidence in their assessment and management of your cancer or treatment related concerns?	4.75

^a^1 = poor; 2 = fair; 3 = good; 4 = very good; 5 = excellent

Although the majority of participants (39/60; 65.0%) indicated that the COVID-19 pandemic did not affect their decisions to present to the hospital whilst unwell, most (33/60; 55.0%) responded that the availability of the CUAC service *more likely* or *much more likely* affected their decision to present to hospital whilst being unwell during the COVID-19 pandemic. The majority of participants (46/60; 76.6%) *agreed* or *strongly agreed* that during the pandemic, it was more convenient to attend the CUAC rather than the ED and they felt safer attending the CUAC compared to ED (48/60; 80%). Thirty-five participants (58.3%) *agreed* or *strongly agreed* they were worried about attending the ED during the pandemic. Participants who previously attended the ED were asked to compare their ED experiences with the CUAC service (Table 4).

**Table 4:** Comparison of ED experiences with the Cancer Urgent Assessment Clinic service

How would you rate the CUAC compared with ED in terms of:	Poor or fair		Good		Very good or excellent		^**a**^Mean score
	***n***	(%)	***n***	(%)	***n***	(%)	
Ease of access to the service?	3	(5.0)	8	(13.3)	30	(50.0)	4.02
On arrival at the hospital, how quick you were assessed and/or treated?	5	(8.3)	5	(8.3)	33	(55.0)	4.07
Overall experience of the ED for assessment and management of cancer or treatment-related concerns?	7	(11.6)	4	(6.7)	32	(53.4)	3.84
Satisfaction of the outcome from your presentation to the ED?	6	(10.0)	6	(10.0)	31	(5.7)	3.84

^a^1 = poor; 2 = fair; 3 = good; 4 = very good; 5 = excellent

Responding whether they had a preference between the CUAC and ED for the assessment and management of cancer and treatment related concerns, 44 chose the CUAC, one the ED and seven had no preference. Responses to open-ended questions showed that patient satisfaction with the service was overwhelmingly positive, particularly with regards to ease of access, time to treatment following arrival, confidence in their assessment and treatment of their cancer or treatment related concern, and likelihood of presenting to the hospital when unwell during the pandemic.

## Discussion

The results showed that 166 of the 400 telephone triaged patients avoided going to the ED as a result of the CUAC model of care. There was also a significant reduction in ED short-stay utilization by patients with cancer during the period of CUAC operation compared to the same period the previous year. Patients reported satisfaction with ease of access to unscheduled care needs and management of disease and treatment related toxicity.

The overall aim of the CUAC service was to offer patients with cancer an alternative to ED presentations. The research conducted at Canberra Hospital showed a 24% reduction in cancer-related presentations to the ED as a results of a Cancer Rapid Assessment Unit [25]. Our study failed to demonstrate a reduction in cancer-related presentations; however, it did show that up to 76.1% of patients referred to the CUAC service potentially avoided an ED presentation. The avoidance of the ED by patients with cancer during the COVID-19 pandemic is a strategy recommended by expert groups through the application of screening algorithms or questionnaires [29, 30]. Whilst the CUAC model of care was initially implemented to reduce the risk of exposure to COVID-19 in the ED of patients with cancer, the evaluation demonstrates that the CUAC model is feasible and an efficient model of care for the management of disease and treatment-related concerns in general.

There was a reduction in the number of cancer patient admissions to the ED short-stay unit, potentially due to the CUAC service model as patients who needed short courses of intravenous antibiotics or observations for managing adverse effects caused by SACTs were managed by the CUAC. Indeed, most of the patients managed by the CUAC team presented with general and systemic complaints of fever and pain. A population study conducted in the USA showed that the top three reasons for ED visits were pain, respiratory distress, and gastrointestinal issues [31]. Gastrointestinal/Urinary were the second highest presenting complaints in our study whereas patients with respiratory distress would have been referred to the ED for COVID-19 testing.

Patients’ satisfaction with the CUAC model of care was high, and they reported higher confidence in their care compared with visiting the ED. Patients rated greater satisfaction with ease of access to specialist assessment and management afforded by the CUAC, consistent with the literature on similar models of care [25]. Also, the majority of patients in our study reported that it was more convenient to attend the CUAC rather than the ED. The primary method of referral was patient/family member-initiated contact via the CUAC telephone triage line, highlighting that telehealth is convenient for patients. Systematic reviews and meta-analyses of telehealth interventions on the quality of life of patients with cancer or cancer survivors showed that telehealth was as effective in improving quality of life as usual care for cancer treatments [32, 33].

Our study had several limitations: it was conducted at a single location which limits the generalizability of the results. ED presentation patient numbers are based on the admitting specialty which could have been the emergency medicine team and not the cancer team. The model of care was implemented during the COVID-19 pandemic and results may differ in non-pandemic circumstances. The survey was completed by a small sample of patients with potential for bias towards treatment by cancer team members who are known to patients compared to ED clinicians.

## Conclusion

The CUAC model of care was implemented to provide access to timely assessment and treatment for patients with cancer experiencing acute disease or treatment-related complications during the COVID-19 pandemic. Whilst initially being implemented to reduce the risk of COVID-19 exposure, this evaluation demonstrated the CUAC model was an efficient and potentially cost-saving model of care for the management of cancer patients with mild to moderate severity of disease and treatment-related concerns. Patient satisfaction with the service was overwhelmingly positive regarding ease of access, time to treatment, confidence in assessment and treatment of cancer related concerns, and likelihood of presenting to hospital when unwell during the pandemic. This evaluation has shown that this model would be feasible and efficient long term to enhance the existing model of emergency care of oncology patients, beyond the pandemic. The CUAC model of care was discontinued due to withdrawal of ongoing funding; however, alternative funding was subsequently secured to build on the model based on the evidence from this study.

## Data Availability

N/A.
